# Optimization of black soldier fly (*Hermetia illucens*) artificial reproduction

**DOI:** 10.1371/journal.pone.0216160

**Published:** 2019-04-30

**Authors:** Bertrand Hoc, Grégoire Noël, Joachim Carpentier, Frédéric Francis, Rudy Caparros Megido

**Affiliations:** Functional and Evolutionary Entomology–Gembloux Agro-Bio Tech (University of Liège), Gembloux, Belgique; Universita degli Studi della Basilicata, ITALY

## Abstract

The black soldier fly (BSF), *Hermetia illucens* (L., 1758) (Diptera: Stratiomyidae), is an endemic fly species from the tropical, subtropical and warm temperate zones of America. This saprophagous species relies on its environment where it finds the decomposing matter for the larvae to grow. The polyphagous diet and the macronutrient quality (mainly lipids and proteins) of these larvae make them excellent candidates for various applications such as waste and organic material management, incorporation in animal feed or alternative energy source. Although rearing development in temperate regions requires artificial processes to continuously produce high quality eggs and larvae, few studies have been conducted on the mating and oviposition processes governing *H*. *illucens* reproduction. Research conducted in semi-artificial rearing conditions showed that the number of mating varied according to the season. It has been speculated that this behavior could be due to differences in the intensity of sunlight caused by the change of seasons. This study aims at evaluating the influence of sex-ratio, density and nycthemeral cycle on *H*. *illucens* reproduction. In order to tackle this issue, an artificial set up for oviposition to collect eggs has been developed. This egg collection system aims at centralize oviposition and simplify eggs collection. Two populations with opposite sex-ratio (male-dominant and female-dominant) were selected. Their respective eggs productions have been evaluated for five breeding densities. Eggs weights varied significantly among the densities for each opposite sex-ratio population and female dominant population produced most eggs weight from 6500 individuals /m^3^. Finally, four nycthemeral cycles (2, 6, 12 and 18h of daily light) were simulated to evaluate the impact of light duration on reproduction. Early oviposition pic associated with a decrease of the oviposition period are shown when *H*. *illucens* are exposed to increasing light duration. These experiments enable improvement of the understanding on artificial reproduction of *H*. *illucens*.

## Introduction

The black soldier fly (BSF), *Hermetia illucens* (L., 1758) is a Diptera of the Stratiomyidae family found throughout the world in tropical and warm temperate regions [1]. This polyphagous species represents a high potential agent for waste management [2]. Larvae are able to consume a wide range of substrates such as agricultural byproducts and animal or plant origin organic waste [3]. This consumption is associated with a strong reduction in organic matter volumes [3, 4], opening the possibility to innovative waste treatment technology through the bioconversion by insects [5]. In addition, the larvae represent a biomass rich in proteins and lipids that can be used as feed for livestock such as fish, poultry and pig [6] or incorporated into biodiesel production [7]. Nevertheless, studies are still needed on the biology of the species in order to evaluate their rearing potential and to develop rearing and reproduction methods [1,5,8]. One of the key steps to mass rear this species is to ensure efficient production of eggs in quantity and quality in order to recover large volumes of organic matter, ensure consistent larval production and maintain progenitors [9].

The reproduction of *H*. *illucens* is divided in two steps (i.e. mating and oviposition) and has only been partially studied [10]. In natural conditions, breeding occurs year-round in the tropics, while it is restricted to a few generations in warm temperate regions [11]. Mating behavior is driven by seasonal variation and more particularly by the days’ duration decrease and its related light intensity [12]. Male territorial behavior has been observed leading to a fight when a male congener gets close while mating effort is undertaken when a female is approaching [11]. Two days after mating, females are ready to lay eggs in an oviposition site if volatile organic compounds are released from surrounding decaying organic matter [13, 14]. Eggs are laid in dry interstices near a moist food resource that will be used as food for future larvae [13]. The reproduction of *H*. *illucens* has been the subject of several studies consisting of biological observation in its natural environment [15], semi-artificial (i.e. regulated greenhouse with sunlight) [1] and artificial breeding methods (i.e. regulated room with artificial light) [16] were developed and optimized [8,10–12,14,17–19]. The reproduction step is conditioned by environmental factors. For instance, Tomberlin and Sheppard (2002) found that mating periods were regulated by light intensity while oviposition was dependent on humidity and temperature [10]. Oviposition was observed to be promoted by temperature above 26°C allowing the development of artificial breeding systems to produce *H*. *illucens* throughout the year in temperate regions [12]. In artificial environment, rearing requires the development of breeding methods in confined environment without sunlight. Zhang et *al*. (2010) obtained mating and oviposition of fertilized eggs with newly-emerged *H*. *illucens* under artificial quartz-iodine lighting while Nakamura et *al*. (2016) also showed that breeding was possible and efficient in low volume cage (0.02 m^3^) under LED artificial lightening [8,16]. Finally, Oonincx et *al*. (2016) indicated that specific wavelengths in LED lightening (LED ratio UV: B: G = 1: 1: 3) could stimulate *H*. *illucens* eyes and ensured reproduction and eggs fertility while Heussler *et al*. (2018) confirmed that LED light was the best lightening to promote flies longevity in small breeding cages compared with fluorescent and halogen lamps [18,19]. These researches highlighted the importance of the breeding methods development in a regulated environment enabling a more in-depth research on environmental and biological parameters that impact and optimize its reproduction. These results could be adapted to a larger scale to develop mass rearing [5]. This study was designed to assess the influence of sex-ratio, density, and nycthemeral cycle on the reproduction of *H*. *illucens* reared in an artificial environment. For this purpose, three successive and complementary experiments were conducted. At first, the evolution of the sex-ratio on colonies raised in a prepupae self-collection breeding model was determined. Subsequently, two opposite sex-ratio populations were selected and bred under different densities. Finally, four nycthemeral cycles (i.e. light duration treatment) have been simulated to evaluate its impact on reproduction.

## Material and methods

### Sex-ratio determination

This experiment was carried out in a transport container (12.04 x 2.33 x 2.38 m, Jindo, Liaoning, China) converted for rearing of *H*. *illucens*. The temperature was maintained at 27 ± 1°C with a relative humidity of 60 ± 5%. The *H*. *illucens* colonies used for these experiments are from an experimental rearing of the Functional and Evolutionary Entomology laboratory in Gembloux Agro-Bio Tech (ULiège, Belgium). Three populations of 10 000 individuals (ind.) with an initial weight of ± 0.01g were grown in polyvinyl chloride (PVC) magnification tanks (76.50 x 56.50 x 30.50 cm, Auer Packaging, Amerang, Germany) at a density of 2.35 ind./cm^2^ and fed on brewing byproducts (distiller grain and hop) and carrot peels. Each tank was closed by a lid, a 45° front slope side which ends with an opening connected to a system of self-harvest PVC tube gutter. This rearing facility allows for larvae self-harvest when individuals exit from the feeding substrate during their prepupal stage. This species behavior is conditioned by the presence of moisten substrate. Some water was added daily to promote the progressive prepupae self-harvest. As they left the tanks, the prepupae were separated in batches of 50g. Five grams of prepupae were randomly collected from each batch and placed in a plastic container (17.20 x 11.50 x 6.00 cm, AVA, Temse, Belgium) covered with a mosquito net where the individuals were kept until emergence. After emergence, the imagoes were sexed manually based on genitalia dimorphism using a binocular (NZ.1902P, Euromex, Arnhem, Netherlands) as described by Oonincx *et al*. (2016)[18]. Twenty-four samples (n = 24, total = 72) per population of about 30 ind. were sexed. These results were used to determine the sex-ratio of the batches and to evaluate the evolution of this parameter in each population. After the self-harvest, the remaining prepupae in the magnification tanks were separated by substrate sieving. These sieved prepupae weights were added to the self-harvested prepupae weights to obtain the total prepupae weight produced per tank.

### Density and sex

**Fig 1:**
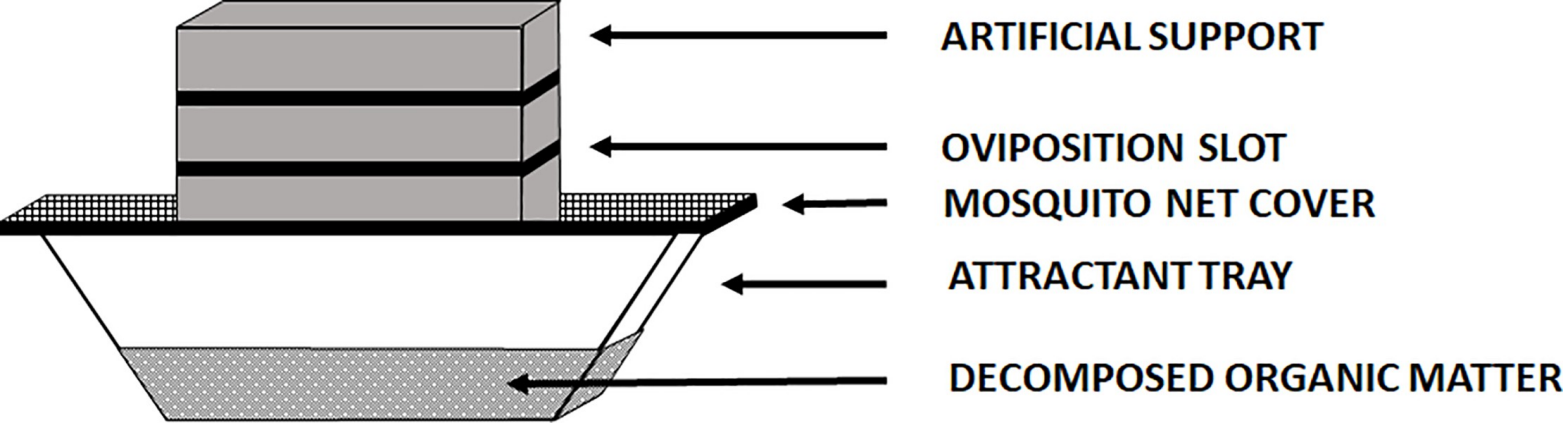
Artificial set up for oviposition to collect eggs.

Fifteen cubic nylon-cages (45.00 x 45.00 x 45.00 cm, Bugdorm, Taichung, Taiwan) were distributed on four shelves inside a room of 5.02 m^2^. The temperature was maintained at 26 ± 1°C with a relative humidity of 60 ± 5%. Two 80 cm LED strips (380–780 nm) were used at the cage top to maintain a 12h - light: 12h - dark photoperiod with a 40 μmol m^-2^ s^-1^ intensity on the cage floor (HD 2302.0, Delta Ohm, Caselle di Selvazzano (PD), Italy). Two progenitor populations with an opposite sex-ratio (i.e. number of male (M) divided by number of female (F) as M/F) were selected at the prepupal stage based on the results found in “2.1 Sex-ratio determination”: sex-ratio of 1.80 as male-dominant (M>F) and sex-ratio of 0.64 as female-dominant (F>M). Five breeding densities were compared in triplicate experiments (n = 3, total = 15) for each sex-ratio: 500 ind./m^3^–2500 ind./m^3^–4500 ind./m^3^–6500 ind./m^3^–8500 ind./m^3^. A moisten sponge placed in a plastic cylinder full of water was used as a water dispenser. An artificial set up for oviposition (i.e. egg collection system) was used to collect eggs (Fig 1). It consists of three pine cambium planks (12.00 x 4.50 x 4.00 cm) held by an elastic band and spaced by screws. The system was placed on a plastic container (17.20 x 11.50 x 6.00 cm, AVA, Temse, Belgium) topped by a mosquito net to prevent egg laying in the laying attractant (Fig 1). This attractant was made of 250 g of 7 days old fermented carrot in a plastic container. The egg collection systems were emptied out every day by separating the cambium planks and by collecting the eggs using a cutter blade. The eggs were weighed on a precision scale (STX223, OHAUS Scout, Parsippany, USA) and summed to obtain the eggs weight per cage. This amount per cage was divided by the number of females to determine the fertility (i.e. eggs weight/female). Other reproduction parameters were recorded: the pre-oviposition period (i.e. duration between first emergence and first oviposition), oviposition period (i.e. duration between first and last oviposition) and oviposition pic (i.e. day with the highest eggs weight from first day-oviposition).

**Fig 2:**
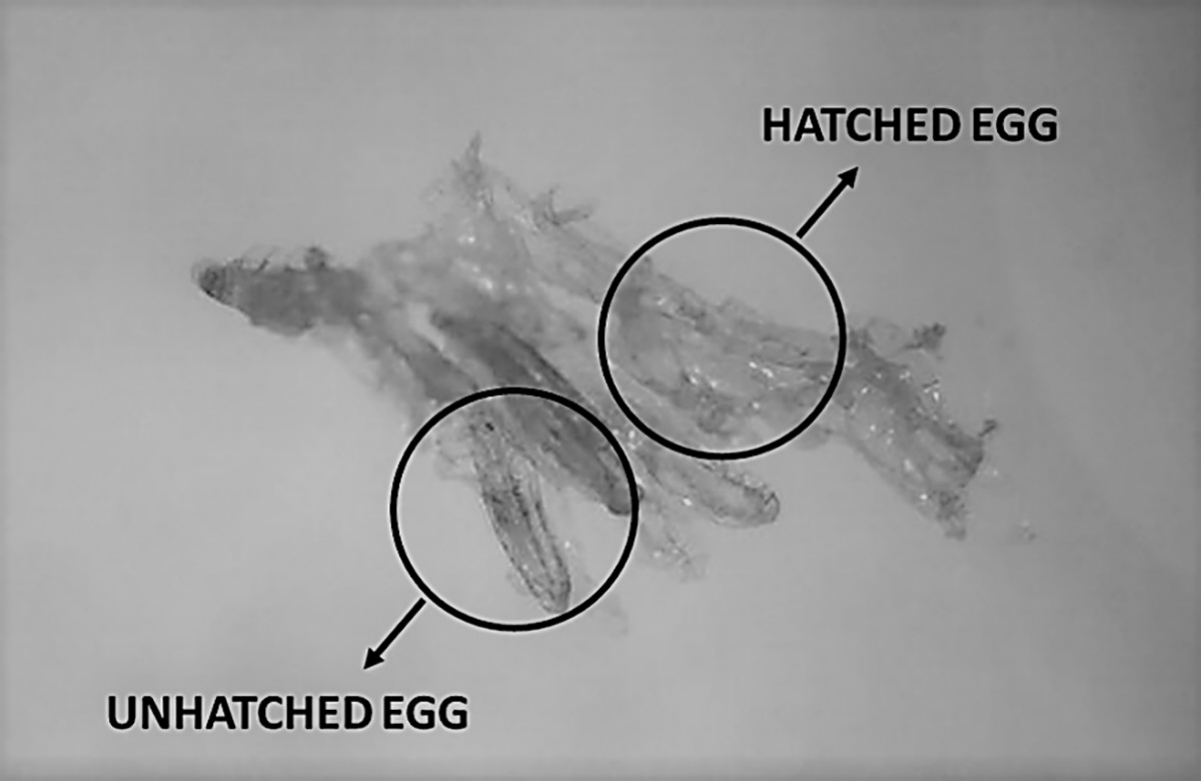
Eggs after hatching.

Following the egg collection, a hatching rate was calculated. As it was not possible to count all the eggs of an egg-laying without damaging them, the relationship between the weight of the entire clutch and the weight of an egg was used. Previously, the average egg weight (0.025 ± 0.003 mg) was determined using a precision scale and by counting the precise egg number of twenty very small batches (n = 20) using a binocular. The average egg weight obtained was similar to those reported by Booth and Sheppard (1984) (0.027 mg) and Kim *et al*. (2008) (0.024 mg) [15, 20]. To estimate the hatching rate, three egg samples per cage were randomly collected at oviposition pic (n = 9 per treatment), were weighed and were kept in a plastic box (92.00 x 66.00 x 50.00 mm; AVA, Temse, Belgium) till hatching. Two days after hatching, the samples were placed under a binocular and unhatched eggs were counted (Fig 2). The difference between the number of eggs sampled (total weight of the egg-laying/ average weight of an egg) and the unhatched ones represents the hatching rate.

### Variation of the illumination period

The third experiment evaluated the light duration influence on the reproduction. A predominantly female sex-ratio colony was selected and dispatched in 12 cubic nylon-cages (45.00 x 45.00 x 45.00 cm, Bugdorm, Taichung, Taiwan) with a 6500 ind./m^3^ density. Cages were isolated in dark conditions by batch of three (n = 3). Cage batches were submitted to four different daily illumination periods of 2, 6, 12 or 18 hours. The egg collection system and the reproduction parameters monitoring protocol presented previously in 2.2 were used.

### Statistical analyses

All analyses were conducted with the Minitab software (version 18 for Windows, State College, PA, USA). The accepted level of significance was 5% in all analyses (i.e. reject null hypothesis). The results were presented as the mean and the standard error of the mean (±SE) and the graphs were performed using R Core Team (2018). Grey shade areas in the graphs indicate 95% confidence interval region computed from the means.

#### Sex-ratio

For the sex-ratio determination, three independent *H*. *illucens* populations (A, B, C) were defined. Using linear regression model, relation between male ratio (dependent variable) and self-harvested prepupae weights (independent variable) for each population was tested. Linear regression was also validated graphically in examining non-constant variance and normal distribution of residuals.

#### Density and sex

Two-way analysis of variance (ANOVA) tests followed by Tukey’s Post-hoc tests were used to evaluate the influence of two factors (i.e. density and the sex-ratio) on eggs weight per cage and fertility (i.e. eggs weight per female) as the assumptions of two-way ANOVA were met. For other parameters (i.e. pre-oviposition period and oviposition period, the oviposition pic and the hatching rate), the ANOVA assumptions were not met. Consequently, Mann-Withney tests (U test) for the two sex-ratio populations (M>F and F>M) and Kruskal-Wallis tests (H test) followed by Mann-Whitney comparisons of specific samples pairs for the six density treatments were used. Two linear regression model, relation between density (independent variable) and eggs weight per cage (dependent variable) for the two sex-ratio populations (M>F and F>M) were tested. We also validated graphically linear regression in examining non-constant variance and normal distribution of residuals.

#### Illumination

One-way ANOVA were used to evaluate the influence of one factor (i.e. light duration) on eggs weight per cage, fertility and oviposition period. The same post-test and ANOVA assumptions as above were tested. The parameters, which did not meet the ANOVA assumptions (i.e. pre-oviposition period, oviposition pic and hatching rate) were analyzed by a Kruskal-Wallis test (H test) followed by Mann-Whitney comparisons for the four light durations treatments. Natural logarithm fitting curve showing best determination coefficient (i.e. variance proportion explained by the model; R^2^) was used to explain the relation between light duration (dependent variable) and eggs weight per cage (independent variable).

## Results

### Sex-ratio determination

**Fig 3:**
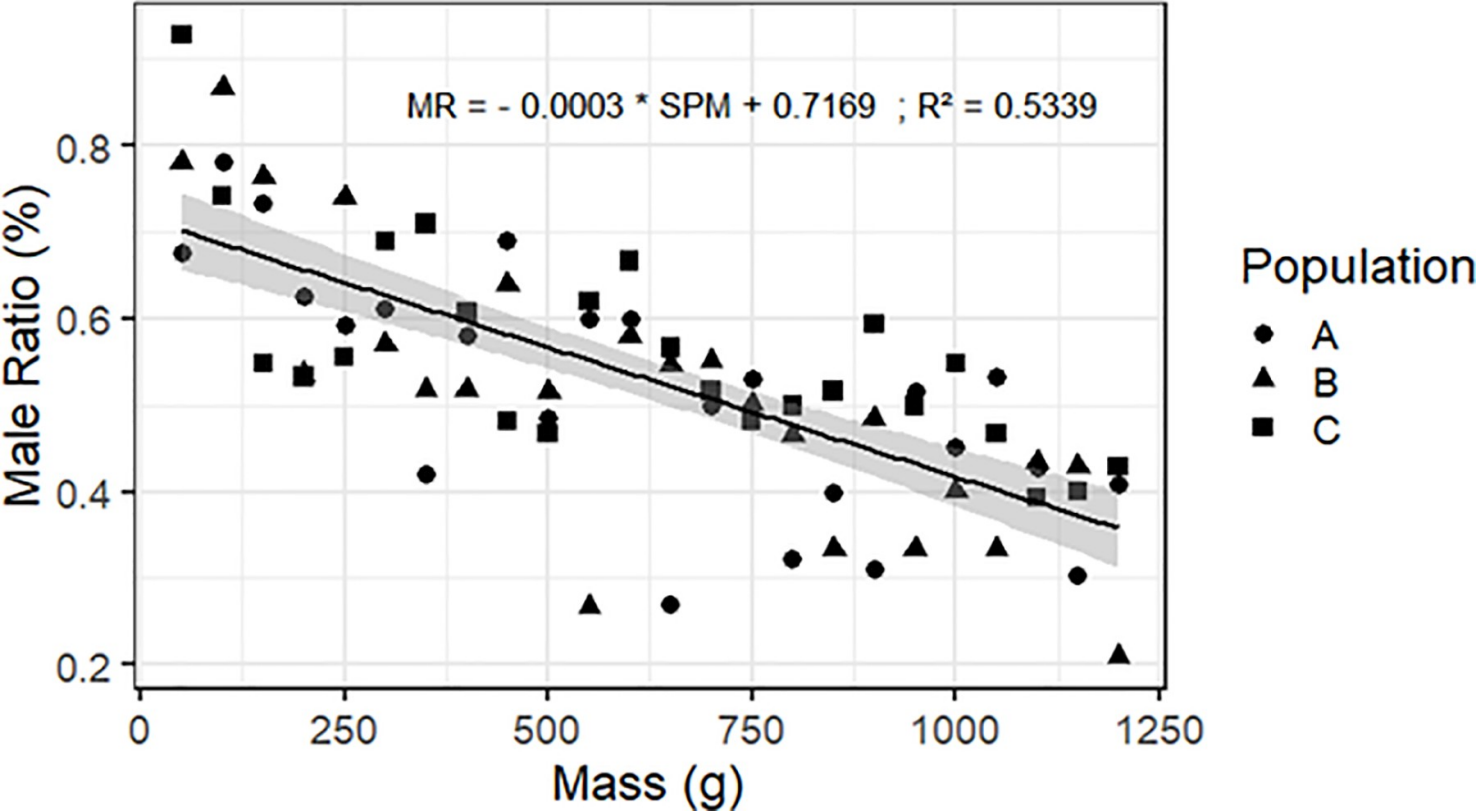
Sex-ratio model. Relationship between male ratio (MR; %) and self-harvested prepupae mass (SPM; g): MR = 0.7169–0.0003 * SPM (F = 80.19, df = 70, P < 0.001, R ^2^ = 0.5339).

The relationship of the male ratio to self-harvested prepupae weight of the 3 populations was described by a linear model. This linear model showed a highly significant decrease for each increment of 50 g of the 3 independent prepupae populations (Fig 3).

The sexually balanced population (male ratio = 0.5) is reached at 700g of self-harvest prepupae or 45% of the total prepupae produced per magnification tank (1561.9g ± 11.1).

### Density and sex

The pre-oviposition periods did not vary significantly between the densities and between the opposite sex-ratio populations (M> F; F >M) (Table 1). The oviposition periods were significantly shorter for the lowest density (500 ind./m^3^) comparatively to the higher ones (2500–4500–6500–8500 ind./m^3^) but did not vary between the populations with sex-disparate proportion. The oviposition pic occurred around the 9^th^ day for all densities but was significantly influenced by the sex-ratio (respectively 8.8^th^ and 10.3^th^ day for the M>F and F>M population). Variance analysis indicated that the fertility was significantly influenced by the sex-ratio (22.5mg ± 0.8 for the M>F population vs 16.6mg ± 1.0 for the F>M population). For eggs weight per cage, the variance analyzes indicated that there was an interaction between density and sex-ratio on this parameter. The data were therefore analyzed separately for each treatments condition (i.e. sex-ratio and density). Eggs weight per cage comparisons of the sex-disparate populations indicated that they did not vary significantly for the three smaller densities (500–2500–4500 ind./m^3^). For 6500 and for 8500 ind./m^3^, female-dominant population produced the higher eggs weight, respectively 7.6g ± 0.1 (vs 5.2g ± 0.4) and 9.0g ± 0.2 (vs 6.6g ± 0.5).

**Table 1:** Life-history parameters of *H*. *illucens* adults under five breeding densities.

Factors		Breeding Density						
		**500**	**2500**	**4500**	**6500**	**8500**	Statistical analyses	*P*
Pre-oviposition (days)		5.6 ± 0.4a	4.7 ± 0.3a	4.5 ± 0.3a	4.3 ± 0.2a	4.2 ± 0.2a	H_5_ = 8.98	0.062
Oviposition period (days)		11 ± 0.7a	16.5 ± 0.6b	15.3 ± 0.3b	16.2 ± 0.6b	15.8 ± 0.6b	H_5_ = 15.34	0.004
Oviposition pic (day)		9.7 ± 0.7a	9.3 ± 0.6a	9.8 ± 0.9a	9.3 ± 0.8a	9.7 ± 0.7a	H_5_ = 0.36	0.986
		**500**	**2500**	**4500**	**6500**	**8500**	Statistical analyses	*P*
Eggs weight/cage (g)	**M>F**	0.3b ± 0.0a	1.8 ± 0.1b	3.8 ± 0.1c	5.2 ± 0.4d	6.6 ± 0.5e	F_4,10_ = 76,34	<0.001
	**F>M**	0.5 ± 0,1a	2.2 ± 0,2b	4.6 ± 0.5c	7.6 ± 0.1d	9 ± 0.2e	F_4,10_ = 199,51	<0.001
Statistical analyses *P*		F _1,4_ = 7.47 0.052	F _1,4_ = 2.38 0.198	F _1,4_ = 2.33 0.201	F _1,4_ = 43.72 0.003	F _1,4_ = 20.89 0.010	F _1,4_ = 7.47 0.052	
		**500**	**2500**	**4500**	**6500**	**8500**	Statistical analyses	*P*
Fertility (mg)		17.4 ± 0.1a	18.1 ± 0.2a	21.5 ± 0.2a	22 ± 0.1a	20.8 ± 0.1a	F_4,20_ = 2.37	0.087
Hatching rate (%)		97.5 ± 0.4a	93.6 ± 2.2a	95.6 ± 1.0a	96.1 ± 0.8a	92.1 ± 2.1a	H_17_ = 7,65	0,105
		**M>F**	**F>M**	Statistical analyses	*P*			
Pre-oviposition (days)		4.5 ± 0.2a	4.9 ± 0.2a	W_14_ = 198	0.158			
Oviposition period (days)		14.3 ± 0.6a	15.6 ± 0.7a	W_14_ = 193.5	0.105			
Oviposition pic (day)		8.8 ± 0.3a	10.3 ± 0.5b	W_14_ = 176.5	0.018			
Eggs weight/cage (g)		3.5 ± 0,6a	4.8± 0.8b	F_1,20_ = 50,72	<0,001			
Fertility (mg)		22.5 ± 0.8a	16.6 ± 1b	F_1,20_ = 24.31	<0,001			
Hatching rate (%)		96.5 ± 0,5a	93.5 ± 1b	W_44_ = 2330.5	0.023			
		**Breeding Density X Sex-ratio**		Statistical analyses	*P*			
Eggs weight/cage (g)		S		F_4,20_ = 8,17	<0,001			
Fertility (mg)		NS		F_4,20_ = 1	0.430			

**Fig 4:**
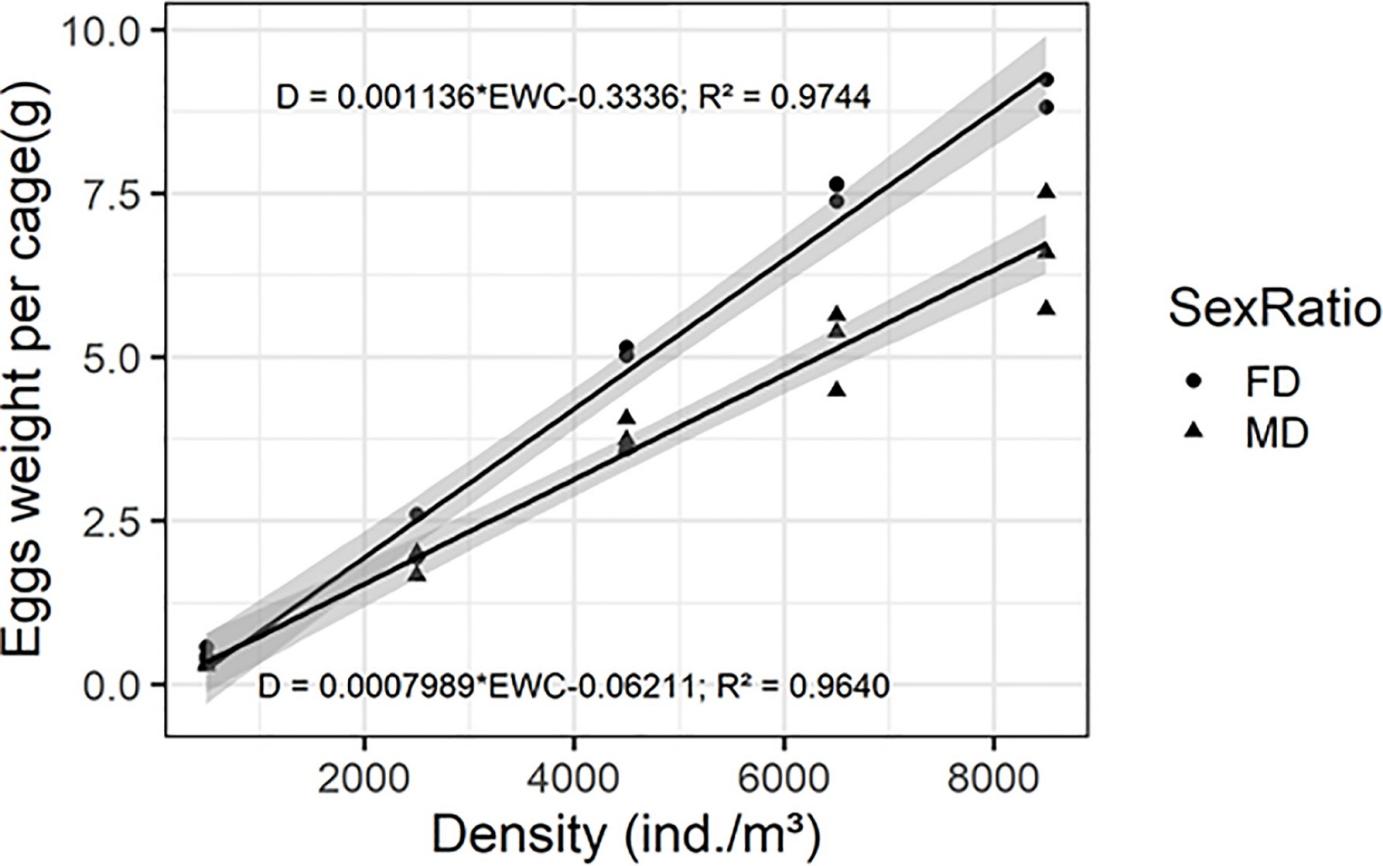
Breeding density model. **Relationship between eggs weight per cage (EWC; g) and five breeding densities (BD; 500–2500–4500–6500–8500 ind./m**^**3**^**) for triplicate experiment.** Higher linear regression line corresponds to F>M sex-ratio (circle points): EWC = 0.0011 * BD– 0.3019 (F = 456.7, df = 13, P < 0.001, R ^2^ = 0.9772). Lower linear regression line corresponds to M>F sex-ratio (triangle points): EWC = 0.0008 * BD– 0.0621(F = 348.4, df = 13, P < 0.001, R^2^ = 0.964).

Two linear relationships were established between the eggs weight per cage and the breeding density, one per population (Fig 4). The hatching rate significantly varied with the sex-ratio (96.5% for M> F and 93.5% for F> M populations) but not according to the densities (Table 1).

### Variation of light duration

For all light durations, eggs were collected from the 4^th^ day after emergence in all cages (Table 2). The oviposition periods varied significantly between the extreme light durations (2–18h) and the intermediate light durations (6–12h). The oviposition pics were significantly influenced by light duration, respectively around the 5^th^ day and around the 3^th^ day for low light durations (2–6h) and long light durations (12–18h). Eggs weight per cage and fertility were significantly lower for the lowest illumination durations (2h). The light duration did not influence the hatching rate (above the 90% for all treatments).

**Table 2:** Life-history parameters of *H*. *illucens* adults under four different light duration periods.

Factors	Light duration					
	2h	6h	12h	18h	Statistical analyses	*P*
**Pre-oviposition (days)**	4a	4a	4a	4a	na	na
**Oviposition period (days)**	13.0 ± 0.6a	16.7 ± 0.7b	15.0 ± 1.0ab	13.3 ± 0,3a	F _3,8_ = 6.04	*0*.*019*
**Oviposition pic (day)**	5.3 ± 0.3a	5.7 ± 0.3a	3.7 ± 0.3b	3.0 ± 0.0b	H_3_ = 9.52	*0*.*023*
**Eggs weight/cage (g)**	4.1 ± 0.9a	6.8 ± 0.5b	7.5 ± 0.7b	7.89 ± 0.21b	F_3,8_ = 23,36	<0,001
**Fertility (mg)**	13.0 ± 1.5a	21.5 ± 0.9b	23.8 ± 1.2b	25.1 ± 0.4b	F_3,8_ = 23.36	*<0*.*001*
**Hatching rate (%)**	98.0 ± 0.4a	96.1 ± 1.5a	96.7 ± 0.1a	94.3 ± 1.6a	H_8_ = 3.95	0.278

**Fig 5:**
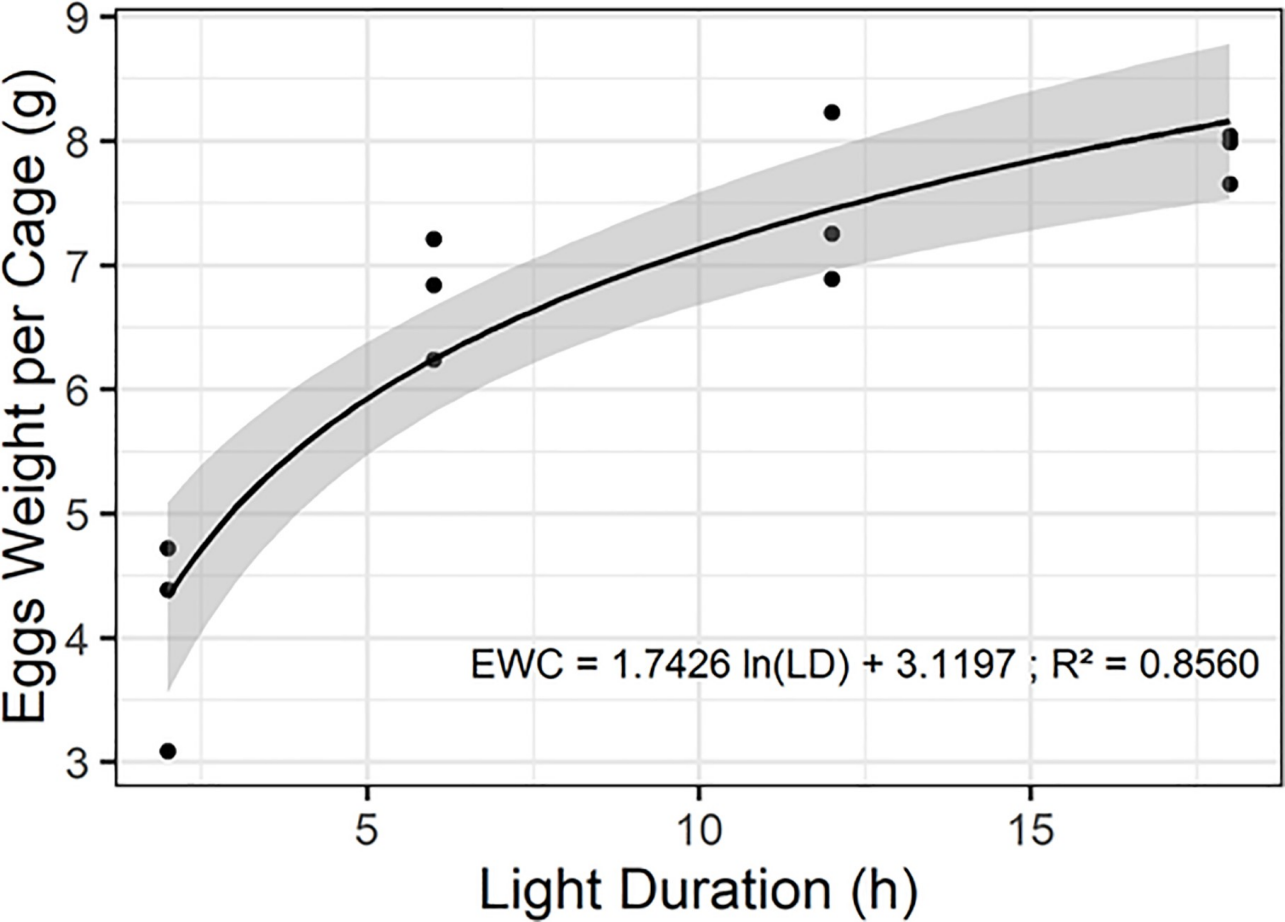
Nycthemeral model. Natural logarithmic relationship between eggs weight per cage (EWC; g) and four light duration treatments (LD; 2h, 6h,12h and 18h): EWC = 1.743 * ln(LD)– 3.120 (F = 59.45, df = 10, P < 0.001, R^2^ = 0.8560).

A logarithmic relationship was established between the eggs weight per cage and the light duration (Fig 5).

## Discussion

Reproduction is a key phase to efficient rearing development of *H*. *illucens*. This life cycle step is currently partially studied and a series of failures in *H*. *illucens* reproduction (e.g. lack of mating and/or low fertility) have been reported in low volume cages exposed to sunlight [1], lit with fluorescent tube [10] or rare-earth lamp [16]. Moreover, the absence of standardized methods further limits the success of reproduction in an artificial environment. This study focused on three parameters conditioning the reproduction: (1) the evolution of sex-ratio on larger colonies, (2) the influence of the density and sex-ratio on the reproduction and finally (3) the impact of the nycthemeral cycle on the reproduction.

### Sex-ratio

The *H*. *illucens* larvae migrate out of substrate at the last immature stage (prepupae) to pupate [4] and can be self-harvested [3, 21]. This species behavior was used to prepupae self-harvest in this experiment. The linear regression applied on male ratio from self-harvested prepupae samples shows a shift of proportion (from M>F to F>M) when approximately 45% of total prepupae weight is harvested. Tomberlin *et al*. (2009) have shown that the larval growth time was sexual-dependent with female harboring a longer larval development time (from 12h to 24h) compared to male [22]. These differences in larval development time could be explained by the fact that female are heavier than males and need more time to develop [13]. According to Tomberlin *et al*. (2009), this weight difference allows females to accumulate higher energy reserves than males, essential for egg production [22]. This model allowed a selection of two opposite sex-ratio populations used in the second experiment on the influence of breeding cages density.

### Density and sex-ratio

The increase in yield of a rearing system is often linked to an expansion in the density of adult individuals. Nevertheless, this change could generate competition between individuals and influence their activity and their reproduction capacity [23]. Park *et al*. (2016) studied the influence of density in high volume breeding cages for *H*. *illucens* in a semi-artificial environment (controlled greenhouse with sunlight) [17]. They found no increase in oviposition and eggs weight for different cage sizes at fixed density. However, an increase in density produced more eggs suggesting that high densities are more productive. In this experiment, breeding densities ranged from 0.0005 flies/cm^3^ (45 flies per cage) to 0.0085 flies/cm^3^ (775 flies per cage) were tested in an artificial environment (controlled room with artificial light). This range includes effective low densities as previously tested by Oonincx *et al*. (2016) (0.0007 flies/cm^3^) but also higher densities as suggested by Nakamura *et al*. (2016) (0.0050 flies/cm^3^) as a determining factor in obtaining mating and fertilized eggs in limited volumes [8, 18]. For all breeding densities tested, the eggs weights per cage increased significantly in the two-opposite sex-ratio populations (M>F: F_4,10_ = 76.34; *P* <0.001 and F>M: F_4,10_ = 199.51; *P* <0.001). These results show that an increase in the breeding density of *H*. *illucens* placed in low volumes ensures and increases reproduction with an improvement of eggs production from 6500 ind./m^3^ for a female dominated population (F>M) (F_1,4_ = 43.72; *P* = 0.003). The two linear models obtained for the two-opposite sex-ratio populations (M>F, F>M) show that we did not achieve the density limiting the reproduction in small volumes of breeding cages (91 cm^3^). Čičková *et al*. (2012) showed that *Musca domestica* L., 1758 (Diptera: Muscidae) could be reproduce efficiently with a density of 0.036 flies/cm^3^ [24]. As *M*. *domestica* flies are approximately 2,66 times smaller than *H*. *illucens*, it could be imagined that a density of 0.013 *H*. *illucens* flies/cm^3^ (or 13000 flies/m^3^) could be efficient.

The fertility was influenced by the sex-ratio, in male-dominant population, each female laid 22.5 mg ± 0.8 of eggs while in female-dominant population 16.6 ± 1.0 mg of eggs were laid (F_1,20_ = 24.31; *P* < 0.001). This could be explained by a lower availability of males in a female-dominant population. Females unable to mate quickly after emergence may reduce the size of their oviposition by partial resorption of their oocytes to fill their metabolic needs [10]. This hypothesis could also explain the delay in oviposition pics’ occurrence between the male-dominant (8.8^th^ day ± 0.3) and female-dominant (10.3^th^ day ± 0.5) populations (W_14_ = 176.5; *P* <0.018). The results on female fertility are consistent with those of Booth and Sheppard (1984) and Tomberlin *et al*. (2002) (respectively, from 29.1 mg to 15.2 mg) and the variability in fertility between experiments could be related to the quality of food during the larval phase [13, 15, 25]. Finally, although fertility is higher for the male-dominant population, the total egg production is significantly higher for female-dominant population and should be favored in terms of eggs yield.

Pre-oviposition periods ranged around 4 days for all densities (H_5_ = 8.98; *P* = 0.062). These durations are comparable to those obtained by Nakamura *et al*. (2016) and Heussler *et al*. (2018) (respectively 4 days and 2–4 days) [8,19]. The breeding density significantly influenced the oviposition periods from 11.0 ± 0.1 days for the lowest density to aroud 15.0 ± 0.5 days for the other densities (H_5_ = 15.34; *P* = 0.004) while the sex-ratio showed no impact on this parameter (W_14_ = 193.5; *P* = 0.105). These durations are longer than those reported by Nakamura *et al*. (2016) showing oviposition period ranging from 7.6 ± 0.8 to 9.4 ± 0.8 days but are close to those of Oonincx *et al*. (2016) and Zhang *et al*. (2010) (respectively 10.0 ± 3.5–16.0 ± 5.3 days and around 19 days) [8, 16, 18]. Differences with Nakamura *et al*. (2016) results could be explained by the progenitors’ selection method as they selected manually individuals that emerged simultaneously for their experiments while individuals emerging randomly from the colonies were used in this experiment leading to a greater heterogeneity of growth between individuals and a potential lengthening of the oviposition period [8]. This method was selected considering the required quantities of individuals for the experiments (6150 flies per experiment) and to reflect the reality of mass breeding.

### Artificial set up for oviposition to collect eggs

Reproductive research on *H*. *illucens* mentions the use of different artificial oviposition supports. Wood [12], paper towel [8], floral foam [17], polypropylene woven sacks [26] can be mentioned but corrugated cardboard is the support generally used and most often cited [1, 10, 13–16, 18, 19, 22]. This support was preferred for the evaluation of the oviposition rate considering that each opening in corrugate cardboard (flute) filled with eggs corresponds to an oviposition [1, 10, 15, 16]. However, this support moistens quickly adding support weighing steps before and after oviposition [15] and several females has been suspected to lay eggs in the same flute, potentially increasing the estimation of oviposition per female [13]. Finally, during their experiments Heussler *et al*. (2018) collected more than 90% of the eggs outside the corrugated cardboard principally on the sponges but also on the cage structures [19].

The oviposition support used in these experiments associated with inaccessible but attractive breeding substrate allowed the centralization of the eggs within the cages and avoid their dispersion. The openings in the device have a large continuous surface allowing continuous oviposition and potentially increase the attraction of congeners’ oviposition as demonstrated by Zheng *et al*. (2013) [14]. The removable nature of the egg-laying surfaces of this support simplifies egg collection and quantification for further applications (e.g. sales, mass breeding or experiments). Finally, this support can be reused since it does not degrade.

### Cycle nycthemeral

The nycthemeral cycle is linked to seasonal changes and conditions the behavior of many insects [27]. For flies, the day length has an impact on fertility whose mating depends on the light [21]. The *H*. *illucens* mating behavior is mediated by sunlight that allows males to detect females and few mating occurs during a short illumination or cloudy day [10]. The artificial light replaces the sunlight and eliminates its variations [16]. In this experiment, the smallest eggs weight per cage (4.1 g ± 0.9) was obtained for the 2 h light duration but did not vary significantly between the three other light durations with a minimum eggs weight of 6.8 g ± 0.5 (F_3,8_ = 23.36; *P* < 0.001). Nakamura *et al*. (2016) report an oviposition number per cage equivalent between 2h sunlight (600 ~ 2000 μmol m-^2^ s-^1^) and 16h LED (47 μmol m-^2^ s-^1^) [8]. Tomberlin *et al*. (2002) showed that the mating number decreased positively with the light intensity [10]. Therefore, it seems that 6 h light duration with a light intensity of 40 μmol m^-2^ s^1^ is a minimum for *H*. *illucens* reproduction in an artificial breeding system.

The oviposition periods decreased with the increase light duration from ~16 days (6h) to ~13 days (18h) except for 2h (F_3.8_ = 6.04; *P* = 0.019). This parameter was previously reported for several light durations, 19 days for 9h [16], 10–16 days for 12h [18], 8–13 days and 7.6 days for 16h of illumination [8,19]. The oviposition pics appeared later for the low light durations (2 and 6h) ~ 5^th^ day and ~3^th^ day for the long light durations (12 and 18h) (H_3_ = 9.52; *P* = 0.023). These oviposition pic timings are supported by several results, 6^th^ and 4–8^th^ days for 16h [8,19] or 13^th^ day for 9h [16]. These results show a reduction in oviposition pic occurrence and oviposition period of *H*. *illucens* when they are exposed to increasing light duration.

The results of these experiments improve the understanding of artificial reproduction of *H*. *illucens*, giving us the ability to optimize experimental rearing models offering high quality and high quantities of eggs. To conclude, a female-dominant progenitor population with, at least a density of 6500 ind./m^3^, could be recommend to maintain an efficient *H*. *illucens* breeding unit under a 6h - ligth: 18h - dark photoperiod. Further studies are still needed to identify the maximum fly density that can be used in a breeding unit (i.e. over 8500 ind./m^3^ following the results of this study or over 13000 ind./m^3^ following the comparison with results founds with *M*. *domestica*) Finally, the use of an artificial oviposition support associated with inaccessible, but attractive, breeding substrate greatly improves the egg collection and avoid their dispersion as well as it allows a more accurate establishment of larval population in future production units.

## Supporting information

S1 Raw data (XLSM)
